# Association of body mass index on sexual function in women with endometriosis: A cross-sectional study

**DOI:** 10.1371/journal.pone.0329110

**Published:** 2026-02-13

**Authors:** Alexandre Vallée, Jean-Marc Ayoubi

**Affiliations:** 1 Department of Epidemiology and Public Health, Foch hospital, Suresnes, France; 2 Department of Obstetrics, Gynaecology and Reproductive Medicine, Foch Hospital, Suresnes, France; 3 Medical School, University of Versailles, Saint-Quentin-en-Yvelines (UVSQ), Versailles, France; Tarbiat Modares University, IRAN, ISLAMIC REPUBLIC OF

## Abstract

**Objective:**

This study aimed to investigate the association between body mass index (BMI) and sexual function, measured by the Female Sexual Function Index (FSFI), in women with endometriosis, adjusting for the number of endometriosis symptoms.

**Methods:**

A cross-sectional study was conducted using an anonymous online survey distributed via social media. A total of 1,586 women with endometriosis participated. BMI was categorized as <25 kg/m²), 25–30 kg/m², or >30 kg/m². Sexual function was assessed using the FSFI questionnaire, with a cutoff score of <26.55 indicating sexual dysfunction. Statistical analyses included multiple linear regression models adjusted for age, couple status, and the number of endometriosis symptoms.

**Results:**

The mean FSFI score was 17 (SD: 9), and the mean BMI was 26 (SD: 6) kg/m^2^. Each one-unit increase in BMI was correlated with a small but statistically significant decrease in FSFI score (β = –0.07; 95% CI: –0.15 to –0.01), suggesting limited clinical importance. An inverse U-shaped association was observed, with both low and BMI > 30 kg/m² linked to lower FSFI scores. BMI > 30 kg/m² was significantly correlated with lower FSFI in women with seven endometriosis symptoms (p for interaction = 0.010).

**Conclusion:**

BMI is significantly correlated with sexual function in women with endometriosis, with both low and high BMI negatively correlating FSFI scores. The association was stronger in women with a higher symptom burden. These findings highlight the need for a multidisciplinary approach integrating metabolic, hormonal, and psychological interventions to improve sexual health among women with endometriosis. Further longitudinal research is needed to explore causality and potential interventions.

## Introduction

Endometriosis is a persistent gynecological disorder marked by the presence of tissue resembling the endometrium outside the uterine cavity. This ectopic tissue remains responsive to hormonal changes during the menstrual cycle, leading to chronic inflammation [1,2]. Due to its diverse symptomatology, endometriosis often mimics other medical conditions, complicating timely diagnosis. Common symptoms include intense menstrual pain (dysmenorrhea), pain during intercourse (dyspareunia), abdominal and pelvic discomfort that may extend to the lower back, as well as pain during urination and gynecological assessments [3–5].

The estimated prevalence of endometriosis ranges between 7% and 15% among women of reproductive age in the general population, with substantially higher rates reported in clinic-based populations such as women with infertility (30–50%) or chronic pelvic pain (≈50%) [5,6]. Moreover, it is diagnosed in nearly half of patients experiencing chronic pelvic pain syndrome [7]. However, because some individuals remain asymptomatic, the true prevalence is difficult to determine, posing challenges to precise epidemiological evaluation [6,8].

In assessing sexual function, the Female Sexual Functioning Index (FSFI), a comprehensive 19-item questionnaire where higher scores denote better sexual function, has been employed [9]. According to findings by Shi et al., women diagnosed with endometriosis exhibited significantly lower FSFI scores in comparison to their healthy counterparts, indicating impaired sexual well-being [10]. Endometriosis can also intrude on various facets of a woman’s life, including her sexual relationships, leading to increased strain within these relationships [11]. Evidence suggests that the symptoms of endometriosis can negatively affect intimate and sexual relationships [12–14]. Sexuality involves both physical and emotional aspects and has a major correlation on a woman’s overall well-being, including her psychological health and relationships.

A number of epidemiological studies reported that women with endometriosis normally have a lower body mass index (BMI) [15,16] or are underweight [17,18]. However, few studies have investigated the relationship between BMI and endometriosis and especially with FSFI score among women with endometriosis. Previous studies showed that the association of BMI and sexual dysfunction in women was inconsistent [19–21]. Although BMI is a widely used and simple anthropometric measure, it is a crude proxy for metabolic health and does not capture differences in body composition, fat distribution, or muscle mass [22]. Nevertheless, BMI remains a pragmatic measure in large-scale epidemiological studies and can highlight trends relevant to sexual health in women with endometriosis. Despite increasing evidence highlighting the significant correlation of endometriosis on sexual health, few studies have explored modifiable factors that may contribute to sexual dysfunction in this population. BMI is one such factor that has been inconsistently correlated with both endometriosis and sexual dysfunction in the general female population. While some epidemiological studies suggest that women with endometriosis tend to have lower BMI or are more frequently underweight, others report an increased prevalence of dysmenorrhea and pelvic pain among obese women with endometriosis. However, the specific relationship between BMI and sexual function, as measured by the FSFI, has been poorly studied in women with endometriosis. Understanding this relationship is crucial, as both underweight and overweight status may influence hormonal balance, inflammatory processes, pain perception, and body image, factors known to affect sexual health. Moreover, identifying whether the association between BMI and sexual function varies according to the severity of endometriosis symptoms may help target interventions more effectively. While both underweight and overweight status have been linked to sexual dysfunction in the general female population, women with endometriosis represent a particularly vulnerable group in whom BMI may play a more critical role. This is because excess or insufficient adiposity can exacerbate disease-specific mechanisms such as chronic inflammation, dyspareunia, hormonal imbalance, and psychological distress, thereby amplifying the correlation of BMI on sexual well-being [23,24]. Understanding this interaction is essential to identify potentially modifiable factors contributing to impaired sexual health in this population. In addition, BMI may not only act as an exposure but can also be influenced by endometriosis itself. Chronic pain, reduced physical activity, fatigue, and psychological distress associated with the disease may lead to weight gain or weight loss, thereby complicating the interpretation of BMI as a risk factor [8,24]. This bidirectional relationship highlights the need to examine BMI in the specific context of endometriosis rather than extrapolating from general female populations. Several psychological, medical, and socioeconomic factors are known to affect both BMI and sexual health in women, such as depression, comorbid metabolic conditions, and medication use. While these influences are well established, little is known about how BMI interacts with the specific symptom burden of endometriosis to shape sexual function. Addressing this knowledge gap may help identify BMI as a potentially modifiable factor within the complex interplay of biological and psychosocial determinants of sexual well-being. Therefore, this study aimed to investigate the association between body mass index and sexual function, as measured by the FSFI, among women with endometriosis, while also accounting for the number of symptoms experienced.

## Methods

### Study design

We designed and conducted a cross-sectional survey using survey software developed by our hospital [25]. The survey was completed anonymously to encourage honest and unbiased responses. A formal a priori sample size calculation was not performed for this study due to its exploratory, cross-sectional design and the use of an open online survey. Recruitment was conducted via social media (Instagram) with a snowball sampling approach, where initial participants were invited to share the survey link within their networks. This strategy aimed to maximize participation among women with endometriosis but did not rely on predefined quotas or structured sampling frames. No a priori sample size calculation was performed, given the exploratory nature of this study. However, a post-hoc power analysis indicated that with 1,586 participants, the study had 79.8% power (α = 0.05) to detect a small correlation size (r = 0.07; Cohen’s d = 0.14), consistent with the observed association between BMI and FSFI.

### Ethical considerations

The study was approved by the Foch IRB: IRB00012437 (approval number: 23-07-05) on 18 July 2023. Written consent was obtained from all participants.

### Recruitment and data collection

The study link was disseminated via social media (Instagram) where participants were asked to forward this link to others they know. All registrants were free to accept or decline the invitation, with no monetary reward received in return. Participants were also informed that they could withdraw at any time. Following internationally accepted ethical codes, respondents were duly informed of the purpose of the survey and were reminded of their participation rights before proceeding to take the survey. A research protocol was conducted to obtain approval from an ethical committee. The distribution of the questionnaire occurred between November 2023 and January 2024 in France on social media (Instagram). We closed the survey link after the workshop ended.

Data were collected through an anonymous, online questionnaire hosted on a secure institutional survey platform developed by our hospital [25]. The survey was designed to prevent multiple submissions from the same participant by restricting access based on device IP addresses. Participants were also explicitly instructed to complete the questionnaire only once and were required to confirm their consent at the beginning of the survey. Furthermore, responses were screened for internal consistency, and entries with contradictory or incomplete information were excluded from the final dataset. These measures aimed to minimize the risk of duplication and ensure data integrity, although we acknowledge that complete elimination of duplicate entries cannot be guaranteed in anonymous, internet-based surveys.

The recruitment strategy via Instagram and social media may have introduced selection bias by favoring younger, digitally engaged, and potentially higher-educated women. Because the survey was anonymous and did not collect detailed geographic or sociodemographic information, the representativeness of the broader population of women with endometriosis could not be assessed.

To minimize duplicate entries, access was restricted by device IP addresses; however, we acknowledge that the use of multiple devices or VPNs could potentially bypass this filter. In addition, both BMI and FSFI were self-reported and therefore subject to recall or reporting bias. These limitations could not be addressed analytically in the present study and should be considered when interpreting the results.

### Inclusion and exclusion criteria

Eligible participants were women aged 18 years or older with a self-reported diagnosis of endometriosis, regardless of disease stage, who provided informed consent and completed the online questionnaire in its entirety. The diagnosis of endometriosis was based on self-report, in line with previous large-scale, survey-based studies in this field [8,25]. Exclusion criteria included participants under 18 years of age. Due to the anonymous and online nature of the survey, clinical confirmation of the endometriosis diagnosis, disease stage, or lesion localization could not be obtained.

Endometriosis was defined exclusively on the basis of self-report, without clinical, imaging, or surgical confirmation. Information on disease stage, lesion type (superficial vs. deep infiltrating), and comorbid conditions was not collected. These factors are known to influence sexual function and therefore represent potential unmeasured confounders in our analysis.

### Questionnaire and measuring instruments

The questionnaire was divided into the following sections:

Sociodemographic questions (couple status, age, educational level, children, BMI level calculated as weight (in kg) divided by height squared (in meters) and BMI categories were defined according to the World Health Organization (WHO) standards as BMI > 30 kg/m^2^, BMI between 25 and 30 kg/m^2^, and less than 25 kg/m^2^ [26].

Questions related to the disease (diagnosis, symptoms, treatment, age of diagnosis etc.)

Symptoms of endometriosis were defined as: pain during sexual intercourse; abnormal or heavy menstruation; infertility; pain during urination during periods; pain during bowel movements during periods, other digestive issues (diarrhea, constipation, nausea); worsening pain over time; pain, particularly excessive menstrual cramps that are felt, and other symptoms, medical treatment for endometriosis, and surgery for endometriosis.

### FSFI questionnaire

We used the validated French version of the Female Sexual Function Index (FSFI), which has been cross-culturally adapted and psychometrically validated for use in French-speaking populations [27].

The FSFI contains 19 items and collects data on 6 domains of sexual function: desire, arousal, vaginal lubrication, orgasm, satisfaction, and pain. For each domain except the pain domain, the item scores range from 0 to 5. Higher item scores indicate better function. Items in the pain domain are coded by a descending scale. To obtain the total FSFI score, the item scores within each domain are added and then multiplied by a correction factor. The resulting scores within each of the 6 domains are added to obtain a total FSFI score. Higher scores reflect better sexual function [9]. A FSFI total score under of 26.55 was considered as to be the optimal cut score for differentiating women with sexual dysfunction to women without sexual dysfunction [28]. For each of the six domains of the FSFI (desire, arousal, lubrication, orgasm, satisfaction, and pain), the sum of the item scores is multiplied by a domain-specific correction factor to standardize the domain scores and ensure that each contributes equally to the total score. The correction factors are as follows: 0.6 for desire, 0.3 for arousal, 0.3 for lubrication, 0.4 for orgasm, 0.4 for satisfaction, and 0.4 for pain [9]. The sum of these weighted domain scores yields the total FSFI score, ranging from 2 to 36, with higher scores indicating better sexual function.

### Statistical analysis

Characteristics of the study population were described as the mean standard deviation (SD) for continuous variables. Categorical variables were described as numbers and proportions. Comparisons between groups were performed using the Mann–Whitney test or t Student test for continuous variables. Pearson’s *χ*^2^ test was performed for categorical variables.

Multiple linear regression models were applied, with adjustment for age, number of endometriosis symptoms and couple, to investigate the relationship between FSFI and BMI.

Adjustment for age, relationship status, and number of endometriosis symptoms was performed to account for known confounding factors that can influence sexual function. Age is a well-established determinant of sexual function, with studies consistently showing a decline in desire, arousal, and overall sexual satisfaction with increasing age [29]. Relationship status is also a critical factor, as being in a stable relationship is associated with higher sexual activity levels and better sexual function scores, while single status may correlate with reduced sexual activity and lower FSFI scores [30]. The number of endometriosis symptoms reflects disease severity and symptom burden, which are strongly linked to sexual dysfunction, particularly due to pain-related mechanisms such as dyspareunia and chronic pelvic pain [31]. Other potential confounders such as disease stage, parity, hormone therapy, psychiatric history, socioeconomic status, smoking, and comorbid metabolic diseases were not available in our dataset. As a result, residual confounding cannot be excluded.

To explore whether the association between BMI and sexual function varied according to symptom burden, interaction term between BMI (both as a continuous and categorical variable) and the number of endometriosis symptoms was included in the multiple linear regression models. The significance of interaction terms was assessed using likelihood ratio tests comparing models with and without the interaction term. Model assumptions were systematically verified. Interaction was examined by including simultaneous BMI and the number of endometriosis sympotms. Relationships between BMI and FSFI were investigated in each subgroup of number of endometriosis symptoms.

The normality of residuals was assessed statistically using the Shapiro–Wilk test. Multicollinearity between independent variables was evaluated by calculating the variance inflation factor (VIF), with all VIF values below 2 (1.05 for BMI, 1.22 for age, 1.01 for beeing in a relationship and 1.80 for symptoms of endometriosis), indicating no significant collinearity concerns.

Statistics were performed using SAS software (version 9.4; SAS Institute, Carry, NC). A *p* value < 0.05 was considered statistically significant.

## Results

### Participants characteristics

1,586 women with endometriosis responded to the online questionnaire, among them, 759 (48%) had children, 1,247 (79%) were in a relationship, 410 (26%) declared smoking tobacco (**Table 1**).

**Table 1:** Characteristics of the study population.

	no sexual dysfunction N = 228		sexual dysfunction N = 1358		
	N/mean	%/SD	N/mean	%/SD	
**Age**					0.043
18-25 years	28	12,28%	209	15,39%	
26-30 years	56	24,56%	266	19,59%	
31-35 years	48	21,05%	279	20,54%	
36-40 years	46	20,18%	289	21,28%	
41-45 years	22	9,65%	204	15,02%	
More than 45 years	28	12,28%	111	8,17%	
Surgery	35	15,35%	204	15,02%	0.898
Education					0.173
Moderate	56	24,56%	331	24,41%	
High	53	23,25%	246	18,14%	
Low	119	52,19%	779	57,45%	
Parity	118	51,75%	641	47,41%	0.225
In a relationship	210	92,11%	1037	76,36%	<0.001
Tobacco	59	26,34%	351	25,92%	0.896
Infertility	65	28,51%	433	31,89%	0.306
BMI level					0.965
>30 kg/m^2^	52	22,81%	312	23,11%	
25-30 kg/m^2^	57	25,00%	346	25,63%	
<25 kg/m^2^	119	52,19%	692	51,26%	
Endometriosis symptoms					0.007
Less than 3	36	15,79%	181	13,33%	
4	45	19,74%	198	14,58%	
5	46	20,18%	203	14,95%	
6	42	18,42%	290	21,35%	
7	55	24.13%	422	31,08%	
More than 7	4	1.75%	64	4.71%	
Desire	7,50	1,56	4,25	2,01	<0.001
Arousal	17,09	2,07	6,78	5,56	<0.001
Vaginal lubrification	17,48	2,55	7,71	6,36	<0.001
Orgasm	13,39	1,67	5,46	4,62	<0.001
Satisfaction	12,03	1,49	6,84	4,65	<0.001
Pain	8,67	1,71	7,22	4,54	<0.001
FSFI	28,51	1,32	14,71	8,40	–
BMI (kg/m2)	25,56	5,62	26,00	5,79	0.287

Characteristics of the population according to the BMI levels are shown in **Table 2**.

**Table 2:** Population characteristics stratified by BMI levels.

	>30 kg/m^2^		25-30 kg/m^2^		<25 kg/m^2^		
	N	%	N	%	N	%	P value
**Age**							**<0.001**
18–25 years	16	4,40%	61	15,14%	158	19,48%	
26–30 years	71	19,51%	56	13,90%	193	23,80%	
31–35 years	93	25,55%	85	21,09%	149	18,37%	
36–40 years	75	20,60%	96	23,82%	160	19,73%	
41–45 years	54	14,84%	67	16,63%	105	12,95%	
More than 45 years	55	15,11%	38	9,43%	46	5,67%	
Parity	208	57,46%	215	53,35%	332	41,14%	<0.001
In a relationship	280	76,92%	337	83,62%	628	77,44%	0.022
Education							<0.001
Moderate	76	20,88%	110	27,43%	199	24,54%	
High	55	15,11%	58	14,46%	184	22,69%	
Low	233	64,01%	233	58,10%	428	52,77%	
Surgery	51	14,01%	42	10,42%	146	18,00%	0.002
Tobacco	110	30,39%	95	23,57%	205	25,47%	0.087
Symptoms							0.002
Less than 3	43	11,81%	75	18,61%	99	12,21%	
4	47	12,91%	62	15,38%	134	16,52%	
5	58	15,93%	44	10,92%	147	18,13%	
6	80	21,98%	76	18,86%	174	21,45%	
More than 7	136	37,36%	146	36,23%	257	31,69%	
sexual dysfunction	312	85,71%	346	85,86%	692	85,33%	0.965
Infertility	139	38,19%	143	35,48%	212	26,14%	<0.001

### Association between BMI and FSFI

Their overall mean for FSFI was 17 (SD: 9) and for BMI 26 (SD: 6) kg/m^2^ (**Table 1**). When considering dichotomization of our study population according to FSFI cutoff (i.e., FSFI<26.55), we observed significant difference for age (p = 0.043), being in a relationship (p < 0.001) and the number of endometriosis symptoms (p = 0.007).

### Adjusted analyses

**Fig 1:**
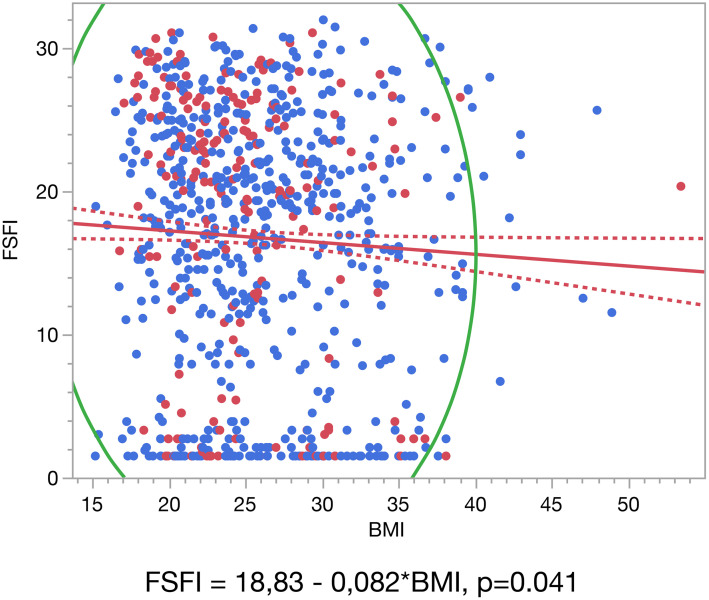
Liner correlation between FSFI and BMI.

As shown in **Fig 1**, we observed a statistically significant negative linear relationship between BMI and FSFI scores (FSFI = 18.83–0.082 × BMI; p = 0.041), indicating that BMI > 30 kg/m^2^ is correlated with lower sexual function in women with endometriosis (**Fig 1**). In practical terms, the negative adjusted beta coefficient observed for BMI (Beta = −0.07; 95% CI: −0.15 to −0.01; p = 0.044) indicates that, on average, each one-unit increase in BMI (kg/m²) is correlated with a 0.07-point decrease in the FSFI score, reflecting a subtle but statistically significant deterioration in sexual function (**Table 3**). Although this correlation may appear modest on an individual scale, its clinical relevance becomes more apparent when considering higher BMI ranges or in women already experiencing substantial symptom burden.

**Table 3:** Multiple regression model for the association between FSFI and BMI with cofounding factors.

Parameters	Estimate	Confidence interval	P value
BMI (kg/m2)	−0,07	(−0.15; −0.01)	0,044
Age			
18–25 years	0,52	(−0.51; 1.53)	0,317
26–30 years	1,02	(0.13; 1.91)	0,024
31–35 years	1,09	(0.21; 1.97)	0,015
36–40 years	−0,71	(−1.59; 0.31)	0,109
41–45 years	−1,58	(−2.60; −0.56)	0,002
More than 45 years	Ref.		
Endometriosis symptoms			
Less than 3	−0,69	(−1.69; 0.31)	0,176
4	0,56	(−0.39: 1.51)	0,247
5	0,94	(0.01; 1.89)	0,048
6	−0,78	(−1.62; 0.06)	0,070
7	Ref.		
Not in a relationship	−3,93	(−4.46; −3.41)	<0.001

### Stratified analyses

**Fig 2:**
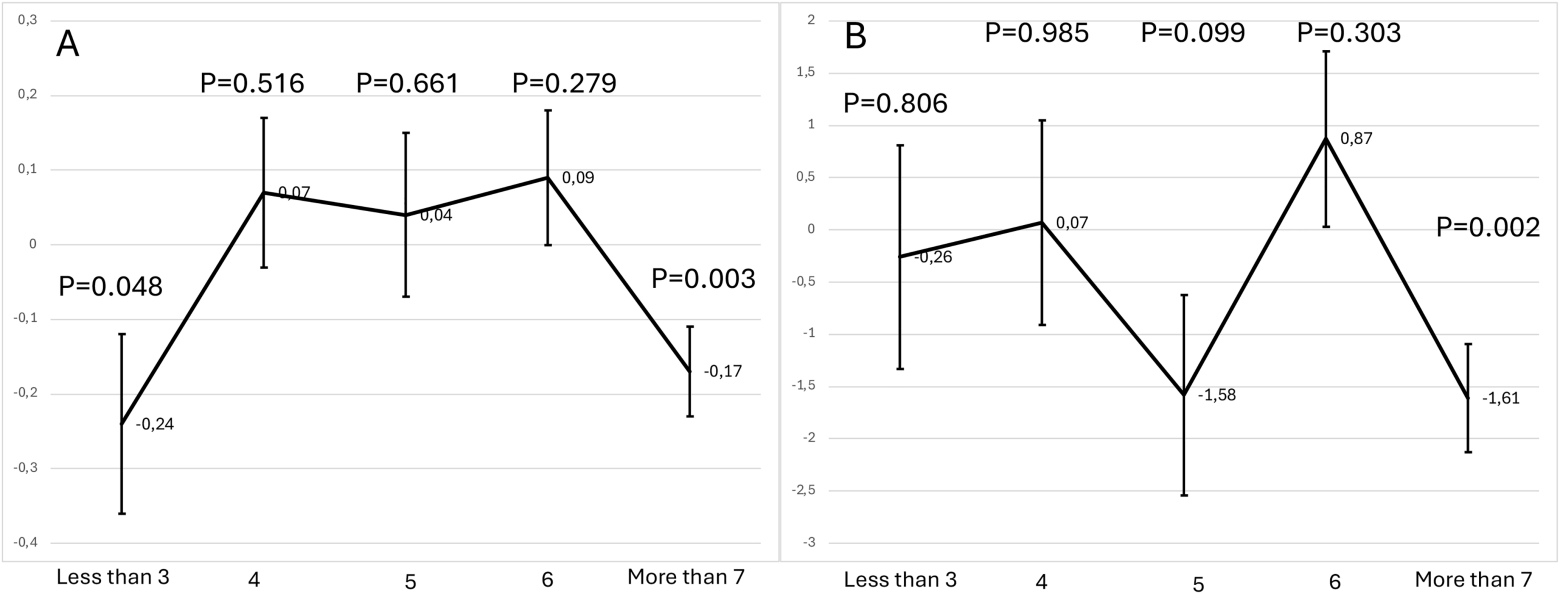
Adjusted beta coefficients with standards errors for the relationship between BMI and FSFI (A) and High BMI level and FSFI (B) according to the number of endometriosis symptoms and adjusted for covariates.

We observed a significant interaction between BMI and the number of endometriosis symptoms (p for interaction, p = 0.020). **Fig 2** illustrates the interaction between BMI and the number of endometriosis symptoms in relation to sexual function. Panel A displays the estimated correlation of BMI (as a continuous variable) on FSFI across subgroups of women categorized by symptom count. Notably, a significant negative association between BMI and FSFI was observed among women reporting seven symptoms (p = 0.003), while no significant association was detected in women with fewer symptoms. Panel B presents similar analyses using BMI categorized as high versus low, confirming that BMI BMI > 30 kg/m^2^ is significantly correlated with poorer sexual function only in women with high symptom burden (p = 0.002). When considering BMI level, after adjustment for confounding factors, BMI > 30 kg/m^2^ was significantly and negatively correlated with FSFI (Beta = −0.71 95% CI: −4.44; −3.39, p = 0.044) (**Table 4**). We observed a significant interaction between BMI level and the number of endometriosis symptoms (p for interaction, p = 0.010). The significant interaction between BMI and the number of endometriosis symptoms (p for interaction = 0.010) suggests that the correlation of BMI on sexual function is not uniform across all women. Specifically, in women reporting seven endometriosis symptoms, a group likely representing those with more severe disease, the detrimental correlation of BMI BMI > 30 kg/m^2^ on sexual function was amplified. In this subgroup, women with a BMI > 30 kg/m² had significantly lower FSFI scores compared to women with BMI < 25 kg/m^2^, even after adjusting for potential confounders. This finding highlights that BMI and symptom burden may act synergistically, compounding the risk of sexual dysfunction. High BMI level was only correlated to FSFI among women with 7 endometriosis symptoms (**Fig 2**). From a clinical perspective, these results emphasize the importance of considering BMI as a modifiable factor, particularly in women with a high endometriosis symptom burden. Addressing both metabolic health and symptom management in a multidisciplinary approach may help mitigate the association of BMI extremes on sexual well-being.

**Table 4:** Multiple regression model for the association between FSFI and BMI level with cofounding factors.

Parameters	Estimate	Confidence interval	P value
Not in a relationship	−3,91	(−4.43; −3.39)	<0.001
Endometriosis symptoms			
Less than 3	Ref.		
4	−0,72	(−1.73; 0.28)	0,157
5	0,55	(−0.39; 1.51)	0,249
6	0,99	(0.05; 1.94)	0,039
7	−0,80	(−1.64; 0.05)	0,063
Age			
18–25 years	Ref.		
26–30 years	0,52	(−0.50; 1.55)	0,312
31–35 years	1,05	(0.16; 1.96)	0,020
36–40 years	1,09	(0.21; 1.98)	0,014
41–45 years	−0,74	(−1.62; 0.13)	0,096
More than 45 years	−1,56	(−2.58; −0.54)	0,002
BMI level			
> 30 kg/m^2^	−0,71	(−4.44; −3.39)	0,044
25–30 kg/m^2^	0,43	(−0.23; 1.09)	0,205
< 25 kg/m^2^	Ref.		

## Discussion

### Summary of findings

The findings of the study indicate a significant negative correlation between BMI and FSFI, suggesting that as BMI increases, sexual function declines. After adjusting for confounding factors such as age, couple status, and the number of endometriosis symptoms, the relationship remained statistically significant. The regression analysis demonstrated that a higher BMI was correlated with lower FSFI scores (Beta = −0.07, p = 0.044). However, the magnitude of this association was small and of limited clinical importance, as a 10-unit increase in BMI corresponded to less than a one-point reduction in FSFI score. This minor correlation suggests that BMI alone is unlikely to be a major determinant of sexual dysfunction, but may act as a contributing factor that amplifies impairment in women already experiencing a high symptom burden. Therefore, while statistically significant, the relationship should be interpreted as clinically modest.Therefore, while BMI may play a contributory role, it is unlikely to be a primary determinant of sexual dysfunction. The clinical importance of our findings lies mainly in highlighting BMI as one among several modifiable factors, particularly in women with high symptom burden, rather than as an isolated predictor of sexual function. However, the study observed an inverse U-shaped association between BMI and FSFI according to the number of endometriosis symptoms.

When examining BMI categories, the study found that women with a BMI > 30 kg/m² had significantly lower FSFI scores compared to those with a moderate or low BMI. This correlation was particularly pronounced among women with seven symptoms of endometriosis. The interaction between BMI level and the number of endometriosis symptoms was significant (p = 0.010), indicating that the association of BMI > 30 kg/m^2^ on signification reduction in sexual function was more pronounced in women experiencing a greater symptom burden.

### Comparison with literature

Our results align with previous studies reporting poorer sexual function in women with endometriosis [14,32] and support findings suggesting that obesity exacerbates this problem [19,20]. However, our study extends current knowledge by demonstrating that this association is modified by symptom severity, which has been less explored. Some discrepancies exist in the literature, with studies reporting no significant relationship between BMI and sexual function [21]. These inconsistencies may stem from differences in study design, population characteristics, and lack of symptom stratification, which our study addressed.

### Biological mechanisms

Obesity is known to be associated with higher estrogen levels [33] and systemic inflammation [34], which could exacerbate the symptoms of endometriosis and, in turn, reduce sexual function. While these mechanisms are biologically plausible, they cannot be confirmed by our cross-sectional data. Additionally, body image concerns and reduced self-esteem associated with a high BMI may further contribute to sexual dysfunction in affected women [35]. Adipose tissue plays a critical role in hormone metabolism, particularly in estrogen production [36,37]. In women with a BMI > 30 kg/m^2^, excess adipose tissue leads to increased aromatization of androgens into estrogens, resulting in higher circulating estrogen levels [38]. This estrogen excess may further stimulate the growth and inflammatory response of ectopic endometrial tissue, worsening endometriosis symptoms such as pelvic pain and dyspareunia [39,40].

Obesity is a pro-inflammatory state characterized by elevated levels of inflammatory cytokines such as tumor necrosis factor-alpha (TNF-α), interleukin-6 (IL-6), and C-reactive protein (CRP) [34]. These inflammatory mediators are also upregulated in endometriosis and contribute to central and peripheral sensitization, leading to chronic pelvic pain, hyperalgesia, and dyspareunia [41]. Chronic inflammation affects vaginal and pelvic muscle function, increasing tension and pain during intercourse, thereby reducing sexual satisfaction [42]. The inflammatory milieu associated with obesity may exacerbate the already heightened inflammatory response in endometriosis [43], leading to a compounded negative correlation on sexual function.

Sexual function depends on adequate genital blood flow, which facilitates arousal and vaginal lubrication [44]. Obesity and metabolic dysfunction are associated with endothelial dysfunction, leading to reduced nitric oxide (NO) availability and impaired vasodilation [45]. This vascular insufficiency can decrease clitoral and vaginal blood flow, leading to difficulties in arousal, lubrication, and orgasm [46]. Furthermore, insulin resistance, common in individuals with high BMI, can further contribute to endothelial dysfunction and reduced genital perfusion, negatively affecting sexual response [47].

Obesity is linked to dysregulation of the hypothalamic-pituitary-adrenal (HPA) axis, resulting in increased cortisol secretion and altered stress responses [48]. Chronic stress and elevated cortisol levels contribute to reduced sexual desire and arousal by downregulating hypothalamic gonadotropin-releasing hormone (GnRH), leading to lower levels of luteinizing hormone (LH) and follicle-stimulating hormone (FSH) [49]. This disruption in the neuroendocrine axis may further decrease ovarian androgen production, which is essential for libido and sexual function [50]. Additionally, endometriosis itself is associated with heightened stress responses [51,52], suggesting that the combination of obesity and endometriosis-related stress may have an additive correlation on sexual dysfunction.

### Psychosocial factors

Both obesity and endometriosis are associated with psychological distress, including depression, anxiety, and body image dissatisfaction [24,53]. Women with BMI > 30 kg/m^2^ often experience lower self-esteem and increased body image concerns, which can reduce sexual confidence, desire, and overall satisfaction [54]. The presence of chronic pelvic pain and dyspareunia in endometriosis further compounds psychological distress, leading to an aversive response to sexual activity [55].

The observed inverse U-shaped relationship between BMI and FSFI indicates that as BMI increases, sexual function (as measured by FSFI) declines, particularly in women with either few or many endometriosis symptoms. This suggests that the correlation of BMI with sexual dysfunction is not linear but varies according to the severity of endometriosis symptoms.

This may be attributed to obesity-related inflammation, endothelial dysfunction leading to reduced genital blood flow, and psychological factors such as body image concerns and reduced self-esteem [56,57]. These factors independently contribute to lower sexual desire, arousal difficulties, and impaired overall sexual satisfaction.

### Clinical implications

In contrast, women with a high number of endometriosis symptoms experience an even greater decline in FSFI scores as BMI increases [10,32]. In this subgroup, the detrimental correlation of BMI on sexual function is amplified by the high symptom burden of endometriosis, resulting in a more pronounced association between obesity and sexual dysfunction. Chronic systemic and localized inflammation from both conditions amplifies pain sensitivity and dyspareunia, further contributing to sexual avoidance [58].

It is also important to consider that low BMI observed in some women with endometriosis may not solely reflect a constitutional factor but could be secondary to disease-related mechanisms. Several studies have suggested that more severe forms of endometriosis, particularly those associated with gastrointestinal symptoms such as nausea, vomiting, diarrhea, and food intolerances, may contribute to reduced caloric intake and unintentional weight loss [15,16]. Furthermore, chronic pelvic pain and systemic inflammation can negatively affect appetite regulation, leading to nutritional deficiencies and lower BMI [6,41]. Hormonal imbalances, particularly involving estrogen metabolism, may also play a role. Endometriosis is associated with altered estrogen pathways and potential disruptions in energy homeostasis [33,37,40]. Moreover, elevated inflammatory cytokines frequently seen in endometriosis may interfere with normal metabolic function and promote catabolic states, further contributing to low BMI [34,41].

In addition to BMI and endometriosis symptom burden, other important confounding factors should be considered when interpreting our results. Notably, the presence of deep infiltrating endometriosis (DIE) lesions and pelvic floor dysfunctions, particularly hypertonic pelvic floor conditions, have been independently associated with impaired sexual function in women with endometriosis. Previous studies have demonstrated that deep lesions, due to their invasive nature and frequent localization near critical anatomical structures such as the rectovaginal septum or uterosacral ligaments, are strongly linked to dyspareunia and overall sexual dysfunction [59]. Moreover, pelvic floor hypertonicity, which often develops as a protective or compensatory response to chronic pelvic pain, can further exacerbate pain during intercourse and reduce sexual satisfaction [60].

An additional factor that may contribute to both symptom severity and impaired sexual function in women with endometriosis is central sensitization, a phenomenon characterized by heightened responsiveness of the central nervous system to nociceptive input. Central sensitization leads to amplification of pain signals, hyperalgesia, and allodynia, often disproportionate to the extent of peripheral lesions [61]. Several studies have demonstrated that women with endometriosis frequently exhibit clinical features of central sensitization, including widespread pain, fatigue, cognitive difficulties, and increased pain sensitivity [62,63]. Notably, central sensitization has been implicated in the pathogenesis of chronic pelvic pain syndromes, contributing to pain persistence even after surgical removal of endometriotic lesions [64].

The presence of central sensitization may partly explain why some women with seemingly limited endometriotic lesions still experience profound dyspareunia and sexual dysfunction. Altered central pain processing not only exacerbates physical discomfort during intercourse but also fosters anticipatory anxiety, sexual avoidance behaviors, and diminished sexual satisfaction [60]. Although our study did not specifically assess central sensitization, future research incorporating validated instruments such as the central sensitization inventory or quantitative sensory testing could help elucidate the role of altered pain processing in mediating both symptom severity and sexual dysfunction in women with endometriosis.

A major strength of this study lies in its large sample size and the ability to examine the interaction between BMI and symptom burden, which has been underexplored in previous research. By addressing both metabolic and gynecological aspects, our findings highlight BMI as a potentially modifiable factor influencing sexual health in endometriosis. This has direct implications for clinical care, as weight management interventions may complement conventional hormonal and pain-oriented treatments. Recent evidence shows that obesity worsens systemic inflammation and endothelial dysfunction, thereby negatively affecting sexual function in women with chronic conditions [34,57]. Furthermore, lifestyle interventions targeting metabolic health and body weight have been shown to improve reproductive and sexual outcomes in women [10,32]. Importantly, psychosocial factors such as body image and psychological distress strongly mediate the relationship between BMI, endometriosis, and sexual function, underscoring the need for integrated management strategies [24]. Thus, our study provides evidence to support a multidisciplinary approach combining metabolic, gynecological, and psychological care to improve sexual well-being in women with endometriosis.

Several unmeasured clinical and psychosocial factors could contribute to the associations we observed. For instance, the presence of deep infiltrating endometriosis lesions may exacerbate dyspareunia and influence FSFI scores independently of BMI [65]. Pelvic floor dysfunction is another relevant determinant of sexual function and may correlate with both obesity and pain symptoms [66]. Similarly, psychological distress, including depression and anxiety, has been consistently linked with impaired sexual function, and its prevalence may vary across BMI strata [67]. Because these variables were not assessed in our cohort, residual confounding remains a possibility and should temper the interpretation of our findings

### Limitations

This study presents several limitations that should be considered when interpreting the findings. First, our sample was recruited via social media (Instagram), which may have introduced selection bias by favoring younger, more digitally engaged, and potentially more health-conscious women. This recruitment strategy could limit the representativeness of our sample, particularly for older women or those with limited access to digital platforms. Consequently, caution is warranted when generalizing these findings to the broader population of women with endometriosis. As a result, the sample may not be representative of the broader French female population with endometriosis. Furthermore, due to the nature of online recruitment and the use of an open survey link, we were unable to calculate a formal response rate, limiting our ability to assess participation bias. French ethical guidelines for anonymous surveys prevented us from collecting geographic identifiers or more detailed sociodemographic information, restricting our capacity to evaluate the regional distribution and diversity of the sample. The reliance on self-reported data for both BMI and FSFI introduces the possibility of recall bias or misreporting, which may have affected the precision of our results. Our cross-sectional design, chosen because it allowed rapid recruitment of a large sample of women with endometriosis outside clinical settings, inherently limits causal inference. Another limitation is the potential for reverse causality. Endometriosis symptoms themselves may influence BMI through mechanisms such as chronic pain, fatigue, reduced physical activity, or altered eating behaviors. This bidirectional relationship complicates the interpretation of BMI as an exposure and raises the possibility that symptom burden not only modifies but also contributes to changes in body weight. Unlike prospective cohort or case–control approaches, it cannot establish temporality or directionality of associations between BMI and sexual function. Therefore, our findings should be interpreted as exploratory and hypothesis-generating, warranting confirmation in longitudinal and clinically validated studies. The questionnaire, designed to be simple and accessible, did not include clinical confirmation of endometriosis diagnosis, information on disease stage, deep infiltrating lesions, or pelvic floor dysfunction, all of which are known to influence sexual function. Similarly, we were unable to assess the role of central sensitization, an emerging factor in pain-related sexual dysfunction. Additionally, our study relied on self-reported measures for both BMI and sexual function (FSFI), which may be subject to recall bias and reporting inaccuracies. Participants may have under- or overestimated their weight, height, or sexual difficulties, potentially affecting the precision of our results. Another limitation is that we were only able to adjust for age, couple status, and symptom count. Other clinically relevant factors such as disease stage, comorbidities, pelvic floor dysfunction, psychiatric history, and medication or hormone use were not available in our dataset. The absence of these variables may have led to residual confounding and therefore limits the robustness and generalizability of the observed associations, as also highlighted in recent studies on endometriosis and sexual dysfunction [24,61]. A further limitation is that the diagnosis of endometriosis was self-reported and not medically verified. This introduces a potential risk of misclassification and internal validity bias. However, symptom profiles in our cohort (e.g., prevalence of dyspareunia, dysmenorrhea, digestive symptoms) were consistent with those described in clinically confirmed populations, supporting the plausibility of self-reported diagnoses. In addition, women reporting a greater number or longer duration of symptoms exhibited significantly lower FSFI scores, consistent with disease severity patterns observed in validated cohorts. These concordant trends provide indirect support for the reliability of self-reported data, although misclassification cannot be entirely excluded.

## Conclusion

In this cross-sectional study of women with self-reported endometriosis, we observed a statistically significant but small association between BMI and sexual function scores. The correlation size was of limited clinical importance, corresponding to minimal differences in FSFI per BMI unit. Thus, while BMI may contribute to sexual dysfunction in the context of high symptom burden, it is unlikely to represent a major determinant of sexual health outcomes. Importantly, our findings reflect correlations only and cannot establish causal relationships or the potential benefits of weight management interventions on sexual health. The study design also imposes notable limitations. Participants were recruited through social media, and endometriosis was self-reported without clinical confirmation or staging, which restricts external validity. BMI was self-reported rather than measured, and relevant confounders, including mental health, treatment history, hormonal therapy, and comorbid conditions, were not captured. Taken together, these factors limit the generalizability of the findings and caution against extrapolating them to all women with endometriosis. Future research should prioritize prospective longitudinal cohorts with clinically confirmed endometriosis diagnoses and staging, more representative recruitment strategies, objective measures of BMI, and comprehensive adjustment for biological, psychological, and treatment-related confounders. Such studies are needed to clarify the directionality and mechanisms underlying the observed correlations and to determine whether BMI plays a meaningful role in sexual health outcomes among women with endometriosis.

### Key messages

-Higher BMI is linked to lower FSFI scores, with sexual dysfunction observed in both women with few and many endometriosis symptoms, highlighting a non-linear association.
-Obesity exacerbates inflammation, hormonal imbalances, and pain sensitivity, worsening sexual dysfunction, particularly in women with severe endometriosis symptoms.
-Targeted interventions addressing metabolic health, pain management, and psychological support are essential to improve sexual function in women with endometriosis across different BMI categories.
